# Sustainable Mortars for Application in the Cultural Heritage Field

**DOI:** 10.3390/ma14030598

**Published:** 2021-01-27

**Authors:** Michelina Monaco, Marianna Aurilio, Anna Tafuro, Mariateresa Guadagnuolo

**Affiliations:** 1 Department of Engineering, University of Sannio, Piazza Roma 21, 82100 Benevento, Italy; monaco@unisannio.it; 2 Department of Architecture and Industrial Design, University of Campania Luigi Vanvitelli, Abbazia di San Lorenzo ad Septimum, Via S. Lorenzo, 81031 Aversa, Italy; marianna.aurilio@unicampania.it (M.A.); anna.tafuro@unicampania.it (A.T.)

**Keywords:** lime mortar, pozzolan, restoration, experimental tests

## Abstract

A large part of the world’s architectural heritage is composed of masonry buildings located in seismic areas, and its vulnerability has been shown by the damage caused by the last earthquakes. Meeting the safety demands of cultural heritage buildings according to the performance-based seismic codes requires a deep knowledge of the mechanical properties of material components. Traditional mortars are among these. However, significant samples of structural mortars cannot be taken from existing masonry walls to perform mechanical tests, but tests can, alternatively, be conducted on samples realized according to traditional instructions for composition. Based on a historical study of mix proportions, this paper presents the results of a mechanical test campaign of traditional mortars. The samples were obtained combining lime and pozzolan according to the proportions derived from ancient treatises. The laboratory tests were performed taking into account three different types of limes, and a discussion involving the results presented in the literature is provided. Besides the contribution to fulfilling the lack of knowledge about the mechanical properties of traditional lime mortars, the test results are good references for on-site preparation of mortars for use in restoration. There is a focus on natural pozzolanic lime mortars, widely used in the Neapolitan area and, in general, in the whole Italian territory.

## 1. Introduction

During the centuries, mortar has been one of the most used building materials, and it was used in combination with natural stones, bricks or artificial blocks to raise the complex buildings of today’s world cultural heritage. The mix of natural pozzolan and lime has been the base of the mortars used in the course of history, and the interest in the field is testified to by several experimental investigations present in the literature [1,2]. Good rehabilitation interventions, especially those performed on cultural heritage buildings, should involve a deep knowledge of the physical and mechanical properties of both the present materials and those to be employed [3,4].

A large part of the world’s architectural heritage is composed of masonry buildings placed in seismic areas, and its vulnerability has been shown by the damage caused by the last earthquakes. Meeting the safety demands of cultural heritage buildings according to the performance-based seismic codes requires a deep knowledge of the mechanical properties of material components [5,6,7]. Traditional mortars are among these. Significant samples of structural mortars cannot, in fact, be taken from existing masonry walls to perform mechanical tests, but tests can be conducted on samples realized according to traditional compositions [8,9].

Tests on small dimension samples of existing mortars and non-destructive tests can give reliable information about the chemical properties of the in-situ materials [10]. The realization of restoration mortars able to satisfy code provisions with regard to mechanical performances and compatible with the existing codes is a problem that has not yet been solved.

The first Italian codes were based on experimental tests by Baronio and Binda [8], which underlined the deep dependence of mechanical properties on the mix proportions and curing methods. Hydraulic mortars’ structural performance is in fact strongly influenced by the presence of humidity during the curing time, so that the actual properties of the existing mortar are also linked to the environment conditions [11,12]. Nevertheless, structural assessments performed according to actual code provisions are based on mechanical properties, for which standards are provided, and material samples have fixed dimensions that are not comparable with the samples available from the in-situ campaigns [13,14]. In some cases, a good correlation between mechanical and physical–chemical results can be obtained, but this is not a general feature [2,15]. This is the reason for the present test campaign, which is based on historical proportions as well as standard and non-standard tests. The correlation with the historical mortars is made by the composition, while the tests are performed on reproduced mortars.

In the last decades, the use of lime mortar as a building material has been strongly reduced in favor of cement mortars, even in the restoration field, where severe damage can be observed after several years of such interventions [16]. The corresponding traditional workmanship has been lost too. In recent years, given the improvements in the technology processes, there has been a sort of recovery of traditional techniques in the cultural heritage field. Lime mortars have been reconsidered for restoration work, especially in combination with pozzolanic materials [17,18].

A large part of Southern Italy’s cultural heritage involves masonry buildings in which pozzolanic mortar plays a key role, so this paper presents a study on its mechanical properties. The first step in the study was the examination of architectural treatises in order to acquire information about the true mix proportions corresponding to the “the rules of the art” at the time of the printed document. The result of a large campaign of laboratory tests on lime mortars is presented in this paper. Based on the historical survey, three distinct lime types were employed to realize the samples [19].

A discussion of the laboratory outcomes obtained by the authors and those presented in the relevant literature is provided [20,21,22,23,24,25,26,27,28,29] together with correlations between the different compositions and mechanical performance of the mortar. The Italian seismic rules for normal [14] cultural heritage buildings [6] are based on minimum values for the mortar strengths, otherwise fixed compositions are demanded. For traditional mortars, there is a great lack of data, especially for pozzolanic ones. Pozzolana is available in all of Southern Italy, and its exceptional capacity for reaction with hydrated lime make it a material with good mechanical strength and water-resistant characteristics [30].

Natural pozzolan can be used in the mortar as it comes from the quarry, while the artificial pozzolan needs industrial treatment to acquire the necessary characteristics [31,32], in particular the hydraulic ones. Largely used by ancient Romans, the first traces of lime mortars date back to 7000 B.C. in what is now Palestine and to 5600 B.C. in what is now Serbia. Roman concrete was in fact enriched with pozzolan until the last imperial period, when several monumental buildings were built; Parts of these are nowadays in use. Vitruvius [33], together with Seneca and Plinius [34,35], was the first source from which to derive knowledge about pozzolanic mortars. Vitruvius’ treatise gives information about mix proportions, while Plinius was the first to underline how pozzolanic mortar quality increased with time. The Romans derived their lime technologies from the Greeks, as is reported in Vitruvius’ famous treatise, and spread their knowledge all around the Empire, so the wide range of Roman lime mortars varies according to the local availability of the aggregates. This is true especially for pozzolanic mortars; when the pozzolan was not available at the construction site, the Roman engineers crushed clay bricks to produce artificial pozzolana. The quality of the mortar obtained with this method is, however, lower than the natural pozzolan mortar for which Roman builders were famous. The Roman high-strength mortar lasted until the Middle Ages, when the technique seems to have been lost. Although the use of pozzolanic mortars continued during the ensuing centuries, especially in the Neapolitan area and in combination with tuff blocks, there is no information about mix proportions in the Renaissance treatises, with the exception of a treatise by Francesco di Giorgio Martini [36,37,38,39,40,41,42]. The development of industrial processes to produce good quality lime induced a revival of the use of lime mortars in monumental constructions. Lime mortars were used in the Royal Palace of Versailles at the end of XVII century. It is not until the beginning of the XIX century and the industrial revolution that systematic studies on the mechanical properties of mortars were made by Vicat [43]. The first classification of pozzolan into natural and artificial is in fact due to Vicat [44]. Unfortunately, Vicat’s treatises do not concern pozzolanic mortars. The treatises of the XIX century [45,46,47,48,49,50,51,52,53] give different compositions for pozzolanic mortars, probably because of the local availability and quality of the pozzolan (Table 1). This brief historical and technological framework is the background to current cultural heritage constructions, in other words, the key to understanding what information about traditional mortars is currently needed. In this paper, the compositions of the mortars reported in the examined treatises are put in relation to their mechanical characteristics by means of a systematic set of laboratory tests.

Enabling cultural heritage buildings to meet safety demands, according to the seismic codes, requires a deep knowledge of the mechanical properties of the manufact as well as the restoration materials [6,14]. Reference values cannot be obtained from in situ campaigns, since it is impossible to take significant samples of structural mortars from existing masonry walls to perform mechanical tests. Moreover, the assessment rules often only take into account cement mortars. Test methods and reference values in the last decades have been developed for cement mortars, which have been largely used in the field of cultural heritage since the first years of last century. In this paper, there is a contribution to addressing the lack of knowledge about the mechanical properties of traditional lime mortars. Moreover, it is shown that mortars prepared on site that respect the classical treatises’ proportions attain code-required mechanical strength and that the curing conditions for hydraulic mortars have a strong influence on the values of mechanical strength. These achievements can be the base for further developments and a reference for builders and designers in the restoration field by including improvements about the on-site curing conditions and time intervals.

## 2. Experimental

As previously noted, the large number of tests performed by Vicat on lime mortars is not useful for this work because the pozzolan is not present as an aggregate in the examined samples [31,53]. The treatises in Table 1 were used as a reference for the mix proportions only, while a thorough discussion is provided comparing the laboratory tests performed by the authors with those present in literature and relative to pozzolanic mortar [54]. Three types of mortar specimens were prepared according to the proportions given in Table 2. The components are by weight. The second and third ratios are commonly used in Southern Italy, especially near Naples, where the B and C compositions define the traditional “Malta Mezzana” (Mizzen mortar) [45]. The volume of water was adjusted taking into account the requirement of a workable material.

Since recent Italian building codes do not consider pozzolanic mortars, specimen dimensions, curing and testing methods have been derived from an Italian set of laws dated to 1939, those currently in force [19] or according to the recommendations in [9].

**Figure 1:**
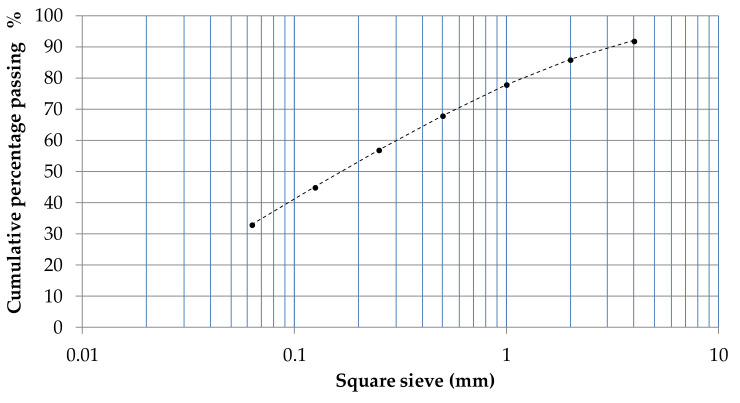
Pozzolan particle size distribution.

All the testing operations were performed at room temperature [19]. The pozzolan sample, which came from the Vesuvian area, was first quartered and then dried to constant mass at 105 °C and sifted. The particle size distribution was assessed by sieve analysis, and the cumulative sieve fractions are reported in Figure 1 as a percent of the total dry weight. It can be noted that the cumulative fraction at 4 mm is 95%, confirming that it is a fine aggregate. The dried pozzolan was mixed with the lime and water according to the proportions in Table 2.

The mixture was placed in the molds at 80% relative humidity, and this last level was maintained until the demolding after 24 h curing. The process was completed by placing the specimens in water to have three different curing times: 28, 60, and 180 days. The geometry and total number of the specimens for every shape are reported in Table 3.

**Figure 2:**
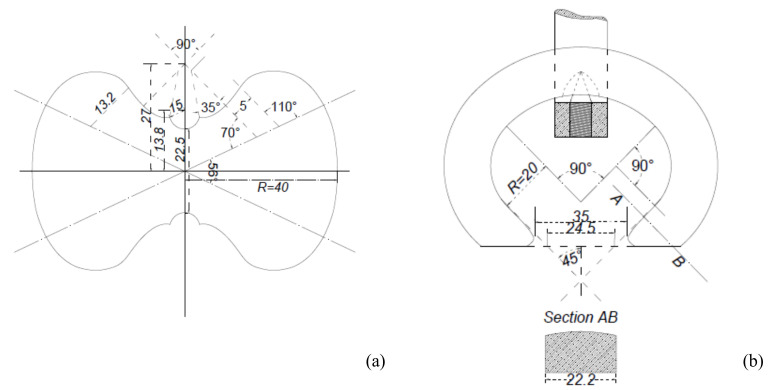
Briquette for the tensile test (**a**) and corresponding grip (**b**); dimensions in mm.

**Figure 3:**
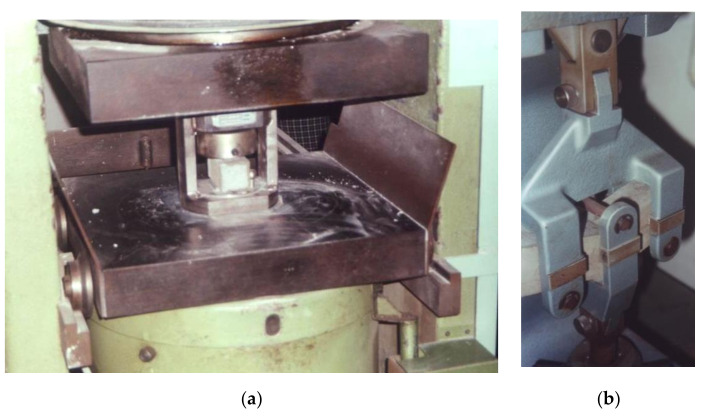
Compressive (**a**) and flexural (**b**) tests.

The double-0 briquettes were employed to perform direct tensile tests, and their dimensions are posted in Figure 2. The minimum cross section of the briquette is 500 mm^2^. The compressive tests on cubic specimens were performed by means of a universal testing machine, C1 class, with a servo-controlled hydraulic actuator with 200 kN force rating (Figure 3a). A loading rate of 300 ± 6 N/s was applied. Tensile and flexural tests were performed by a standard testing machine (Figure 3b) with a loading rate of 50 ± 2 N/s. The direct tensile tests have been performed bearing in mind that the recent developments of diagonal strut methods for the analysis of in plane behavior for masonry are often based on these values [54,55,56]. The choice of large side cubes is due to the exigence of modeling the behavior of rubble masonry in which the inner part of the wall includes a very large mortar element or Neapolitan tuff masonry, which sometimes involve thick horizontal joints. In some cases, it is in fact possible to find tuff masonry walls in which the mortar joints have thickness up to 10 cm.

The dimensions of the cubes were taken after demolding. Currently, the Italian rules require at least three tests for the determination of the mechanical properties of mortar, even that used in the restoration field. In particular [14], two types of mortar are accepted among the commercial products: mortar with prescribed mechanical properties and mortar with prescribed mix proportions, leaving to the designer the responsibility of fixing the composition of the on-site produced mortars. This lack of quantitative recommendations for traditional mortars produced in the restoration yard has induced the development of this research, which is a first step towards guidelines for interventions on cultural heritage with natural pozzolanic mortars.

## 3. Results and Discussion

**Figure 4:**
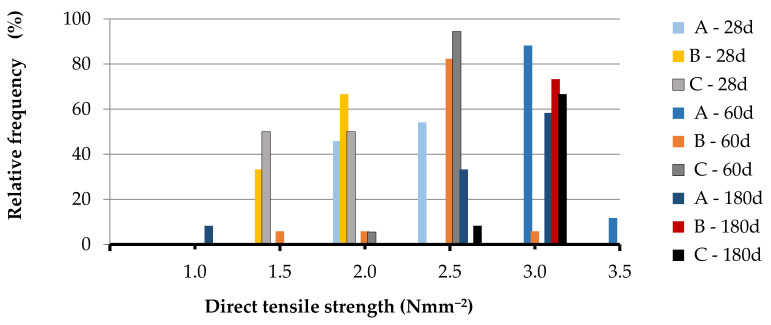
Relative frequency histograms for direct (double o) tensile strength.

**Figure 5:**
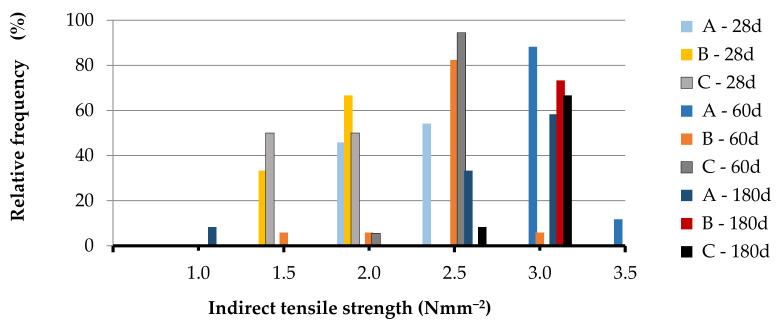
Relative frequency histograms for indirect (three-point test) tensile strength.

**Figure 6:**
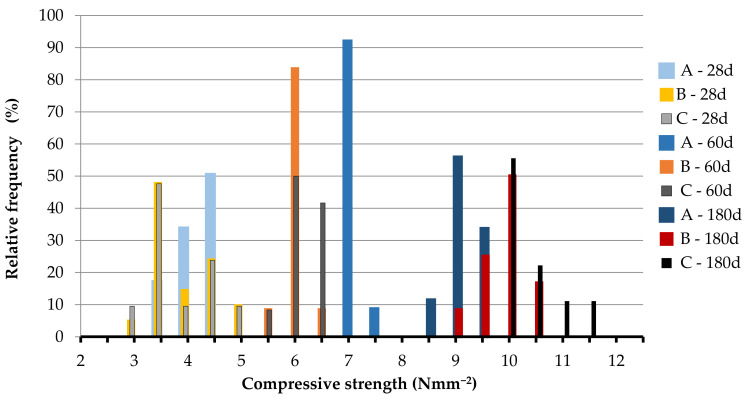
Relative frequency histograms for compressive strength of 70 × 70 × 70 mm^3^.

**Figure 7:**
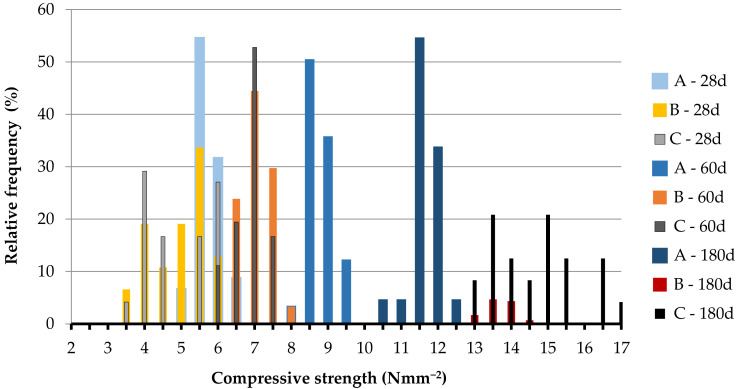
Relative frequency histograms for compressive strength of 40 × 40 × 40 mm^3^.

In all the tests performed, the coefficient of variation (i.e., CV) has a low value so that reference for the discussion is made to the mean values. The details of the single test are reported in the Appendix A Table A1, Table A2, Table A3, Table A4, Table A5 and Table A6 together with the mean value, the standard deviation and the CV of the set, while in Figure 4, Figure 5, Figure 6 and Figure 7 the relative frequency histograms of all the test sets are shown.

Although the number of tests is lower than the minimum necessary to make a complete statistical study, the relative frequency distribution reported in Figure 4, Figure 5, Figure 6 and Figure 7 confirms that the test results can be represented by a Gaussian distribution with small extent of variability in relation to the mean of the data population. This is a starting point to establish the minimum number of tests to be performed for the mechanical assessment of in-situ materials [57].

Italian codes [14] consider a “confidence factor” to be taken into account in the structural assessment to reduce the material strength. In case three, experimental values for a structural material in an existing building are available and the reference value for the structural assessment is the mean value of the experimental data [58], otherwise an increasing confidence factor (depending on uncertainty) is applied to the strength value. The statistical parameter CV, i.e., the percentage ratio between standard deviation and mean value, is considered in the U.S. Federal Emergency Management Agency code [59] to take into account the data dispersion. The mean value of the measured strength can be considered if the CV is lower than 14%. When it is higher, either the number of experimental tests needs to be increased until the CV attains 14% or the mean strength needs to be decreased by the standard deviation value. As it is possible to detect, the CV of every dataset in Appendix A Tables is contained within 14%. In this paper, the mortar composition is designed in reference to historical studies rather than to FEMA 356, where it is made in reference to “surrogate mortar designed on the basis of a chemical analysis of actual mortar samples”. In case of buildings where the extraction of a sample is not possible (walls with decorations or frescoes), treatises on time are the only source.

**Figure 8:**
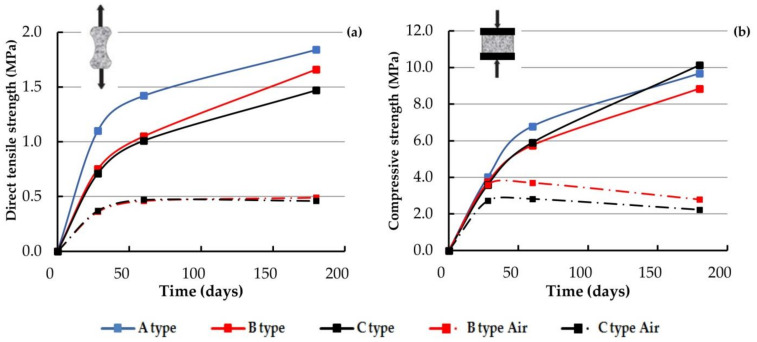
Direct tensile strength of briquettes (**a**) and compressive strength of 70 × 70 × 70 mm^3^ (**b**) versus specimen age.

Figure 8 reports the mean values of direct tensile strength on briquettes and compressive strength of large cubes versus the specimen age for the three examined water cured mortars (continuous line). A comparison with the corresponding values for air cured mortars B and C is provided (dashed line). As can be noted, in the water cured mortars, the increment of strength with age is higher than 80% in every case [60,61], even if the curing time influence on the compressive strength of large cubes is greater than that on the tensile strength. The same behavior is not observed in the air cured mortars since the strength values do not increase with the curing time.

The variation of the corresponding data for water cured and air cured mortars is strongly sensible too. The importance of water curing for natural pozzolanic mortars has been well known since the Vitruvius treatise [33], which states, “Ergo cum tres res consimili ratione ignis vehementia formatae in unam pervenerit mixtionem, repente receptor liquore una cohaescerunt et celeriter umore duratae solidantur, neque eas fluctus neque visa quae potest dissolvere” (“Since, then, three things of a similar nature—tuff, lime and pozzolan—arising from the intensity of the fire, combine in one mixture, as soon as moisture supervenes, they cohere and quickly harden through dampness so that neither the waves nor the force of the water can disunite them”). Recommendations on the correct way of in situ curing of mortars could be found in many XIX century treatises [49] and in the codes of practice of the time [52], where the indications are usually placed in the parts pertaining to masonry building. In fact, old Italian masons used to soak the tuff blocks before use and soak the masonry wall during the following days. These rules of art have unfortunately been lost, although they should be included in the current codes of requirement used in the restoration field.

The A-type mortar is made, in distinction to the other two types, with powdered lime. Its strength rate increment is faster with respect to the other two binders since lime putty needs several months to complete curing. According to the indications of Plinius [34], “In antiquorum aedium legibus invenitur, ne recentiore trima uteretur redemptor, ideo nullae tectoria eorum rimae foedavere” (“In the old building laws is to be found a regulation that no contractor is to use a putty that is less than three years old”). This indicates that at least three years are necessary to obtain a good quality mortar. Recent literature papers have observed a strong increase in strength of lime mortar after several months with regard to the 28 days results [62,63,64,65]; this is the reason for the 180 days test used in this paper. It is possible to observe that the 180 days compressive strength of A-type mortar is lower than that of the C-type mortar, both for large and small cubes.

**Figure 9:**
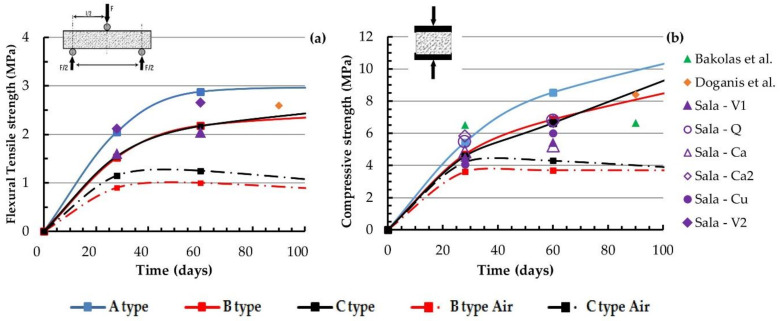
Flexural tensile strength (**a**) and compressive strength of 40 × 40 × 40 mm^3^ versus specimen age (**b**).

In the literature, there is a lack of direct tensile test results, due perhaps to the complex realization of specimens without damage or disturbance. The tests referred to in the literature are mainly standard three-point flexural tests and compressive tests on small cubes, which are obtained as a result of the flexural test, so that a comparison is possible with only these types of tests performed by the authors, reported in Figure 9 [20,21,22,25]. As previously seen, the water cured mortars’ data and the air cured mortars’ data are represented with continuous and dashed lines respectively.

The three-point test gives values of the mortar’s indirect tensile strength higher than those of the direct test. Suitable corrective factors should be involved in the numerical constitutive models when indirect tensile strength is taken into account since direct tensile strength ranges from 40% to 60% of the indirect strength. Figure 9 reports a comparison between the authors’ results and those derived from some experimental papers reporting tests on mortar samples, and only natural pozzolanic mortar tests have been taken into account. The laboratory test data reported in Figure 9 are in the same range as that obtained by the authors and have been made on mortars with similar mix proportions, while those with lower strength have been made with a lower ratio of pozzolana versus lime [24,25,27]. For more clarity and more possibilities for making comparisons, the data relative to the 180 days tests have been skipped in the pictures and are reported in the Appendix A Table A5 and Table A6 only. In these pictures, the data relative to the air cured mortars made by the authors are also reported. It is in general verified that the mortars realized with hydraulic materials (i.e., natural pozzolan) and immersed in water for curing have better mechanical performances compared to the air cured ones.

**Figure 10:**
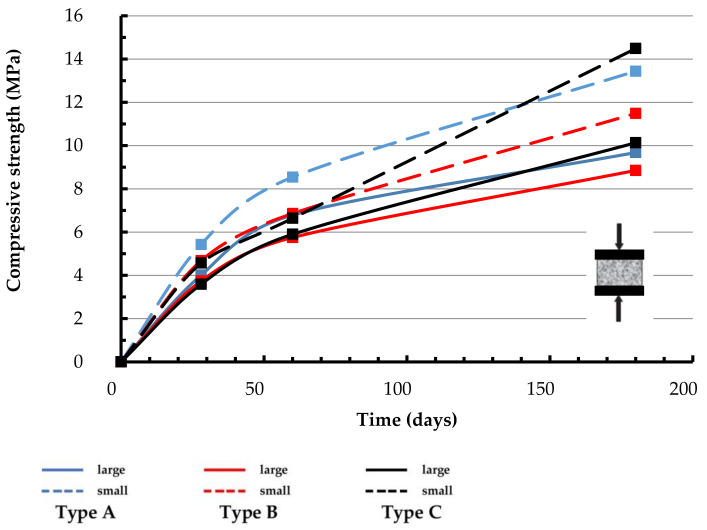
Compressive strength of large (70 × 70 × 70 mm^3^) and small (40 × 40 × 40 mm^3^) cubes versus specimen age.

Figure 10 reports the compressive strength of both the cubic specimen types versus curing time. The confinement effect due to the friction with the machine plates makes the compressive strength of small cubes higher than that of large ones since the dashed curves relative to the first ones overlie at every curing age the continuous curves due to the large cubes. It must be noted that even the lower compressive strength satisfies the requirements of the Italian Standards for lower bound strength of building mortars fixed at 2.5 N/mm^2^ [14].

It must be noted that the mechanical performances of the three types of mortar tested by the authors are somewhat similar, with small differences due to the lime type. In particular, the laboratory slaked putty and the manufactured mortar, independently of the production process, give comparable results in compression and tension range. This feature can be useful in the professional field since the yard realization of a lime putty can be avoided by choosing a manufactured product resulting from a quality process.

## 4. Conclusions

This paper presents the results of a large laboratory test campaign performed by the authors on three different types of pozzolanic lime mortars, whose compositions are derived from architectural treatises. Standard three-point tests, together with compressive tests on small and large size cubes, as well as direct tensile tests have been performed on every type of lime mortar.

The results show that long-term curing increases the mechanical performance of mortars based on lime putty more than those based on powdered lime; this is different from the weeks immediately following the preparation, when a reverse behavior is observed. In general, water cured natural pozzolanic mortars show better mechanical performance compared with mortars made with the same mix proportions but cured in air.

The analysis of the test data encourages the use of lime mortars in the restoration field and the development of suitable guidelines for the relevant professionals. These guidelines should respect “the rules of the art” since the curing conditions, the pozzolan quality and the mix proportions strongly influence the quality of the results.

## Figures and Tables

**Table 1:** Pozzolanic mortars mix proportions (by weight ratios) traced in architectural treatises.

Reference	Period	Pozzolan	Lime	Sand
Vitruvius Pollio [33]	I cent. B.C.	2.00	1.00	
Martini di Giorgio F. [36]	1470	0.50	1.00	
Viviani Q. [45]				
Hydraulic works	1830	1.33	1.00	0.67
Normal	1830	2.00	1.00	1.00
Valadier G. [46]	1831	5.00	1.00	
Quatremere de Quincy [47]				
Strong	1832	3.00	1.00	
Normal	1832	2.00	1.00	
Cavalieri S. Bertolo N. [48]				
Stone wall	1832	5.67	1.00	
Palette walls	1832	3.00	1.00	
Bulk brick masonry	1832	2.33	1.00	
Curtain brick masonry	1832	1.22	1.00	
de Cesare F. [49]				
Normal	1855	2.00	1.00	3.00
Cocciopesto	1855	1.20	1.00	0.60
Constructions in water	1855	1.00	1.00	2.00
Plaster with Santorini earth	1855	5.18	1.00	
Claudel J. & Loroque L. [50]				
Weak	1863	0.80	1.00	1.80
Normal	1863	1.00	1.00	2.22
Strong	1863	1.11	1.00	2.78
Curioni G. [51]				
Weak	1864	0.50	1.00	0.25
Normal	1864	3.00	1.00	
Strong	1864	1.00	1.00	2.00
Levi C. & Astrua G. [52]	1927	3.00	1.00	

**Table 2:** Ratios of Components for Mortars in weight [kg].

Mortar Type	Lime Type	Lime	Pozzolan	Water
A	Industrial hydrated lime	1.00	3.00	1.37
B	Industrial lime putty	1.00	4.17	1.06
C	Laboratory lime putty *	1.00	4.11	1.13

* obtained slaking quicklime in laboratory.

**Table 3:** Geometry and number of specimens.

Shape	Number	Length (mm)	Depth (mm)	Width (mm)
Prism	12	40	40	160
Cube	24	40	40	40
Cube	12	70	70	70
Briquette	12	80	22.5	22.2

## Data Availability

All data are included in the paper.

## Appendix A. Test Data

**Table A1:** Tests on specimens 28 days age (Cube 70 × 70 × 70 and Briquette).

Specimen Number	Compressive Strength 70 × 70 × 70 (N/mm^2^)	Tensile Strength Briquette (N/mm^2^)	Specimen Number	Compressive Strength 70 × 70 × 70 (N/mm^2^)	Tensile Strength Briquette (N/mm^2^)	Specimen Number	Compressive Strength 70 × 70 × 70 (N/mm^2^)	Tensile Strength Briquette (N/mm^2^)
A-Type Mortar			B-Type Mortar			C-Type Mortar		
1A28	4.38	1.10	1B28	3.44	0.73	1C28	3.68	0.71
2A28	4.38	0.98	2B28	2.88	0.71	2C28	3.30	0.71
3A28	4.40	1.28	3B28	3.56	0.80	3C28	3.08	0.65
4A28	4.32	0.96	4B28	3.28	0.75	4C28	3.12	0.61
5A28	4.34	1.14	5B28	3.48	0.73	5C28	3.12	0.74
6A28	4.40	1.22	6B28	3.50	0.69	6C28	2.84	0.67
7A28	4.13	1.18	7B28	3.40	0.70	7C28	2.68	0.59
8A28	4.44	1.28	8B28	3.28	0.69	8C28	3.24	0.71
9A28	4.34	1.22	9B28	3.06	0.61	9C28	3.26	0.59
10A28	4.40	1.10	10B28	3.36	0.73	10C28	3.14	0.73
11A28	4.36	1.16	11B28	3.36	0.75	11C28	3.28	0.51
12A28	4.36	1.04	12B28	3.40	0.69	12C28	3.36	0.59
13A28	3.76	0.90	13B28	4.80	0.73	13C28	3.84	0.80
14A28	3.92	1.14	14B28	4.00	0.86	14C28	4.52	0.69
15A28	4.00	0.94	15B28	4.20	0.78	15C28	4.44	0.88
16A28	3.40	1.12	16B28	4.28	0.88	16C28	4.04	0.75
17A28	3.86	1.14	17B28	3.96	0.78	17C28	4.20	0.80
18A28	3.40	1.10	18B28	4.40	0.77	18C28	4.24	0.73
19A28	3.84	1.16	19B28	4.08	0.88	19C28	3.08	0.88
20A28	3.68	1.12	20B28	4.20	0.77	20C28	4.20	0.84
21A28	3.76	1.08	21B28	4.56	0.78	21C28	4.72	0.77
22A28	3.68	1.06	22B28	-	-	22C28	-	-
23A28	3.20	1.06	23B28	-	-	23C28	-	-
24A28	3.36	1.06	24B28	-	-	24C28	-	-
**AVERAGE**	**4.01**	**1.10**		**3.74**	**0.75**		**3.59**	**0.71**
**ST. DEV.**	**0.40**	**0.10**		**0.53**	**0.07**		**0.61**	**0.10**
**CV%**	**9.98**	**9.09**		**14.17**	**9.33**		**16.99**	**14.08**

**Table A2:** Tests on specimens 28 days age (Cube 40 × 40 × 40 and Prism).

Spec. N.	Ind. Tens. Strength 40 × 40 × 160	Compr. Str. 40 × 40 × 40 (N/mm^2^)	Compr. Str. 40 × 40 × 40 (N/mm^2^)	Spec. N.	Ind. Tens. Strength 40 × 40 × 160	Compr. Str. 40 × 40 × 40 (N/mm^2^)	Compr. Str. 40 × 40 × 40 (N/mm^2^)	Spec. N.	Ind. Tens. Strength 40 × 40 × 160	Compr. Str. 40 × 40 × 40 (N/mm^2^)	Compr. Str. 40 × 40 × 40 (N/mm^2^)
A-Type Mortar				B-Type Mortar				C-Type Mortar			
1A28	2.11	5.76	5.76	1B28	1.52	4.66	4.29	1C28	1.20	3.86	4.11
2A28	2.13	5.82	5.46	2B28	1.57	4.23	4.54	2C28	1.18	3.37	3.56
3A28	2.26	5.64	5.64	3B28	1.58	4.78	4.41	3C28	1.47	4.17	3.80
4A28	2.23	5.58	5.76	4B28	1.62	4.78	4.66	4C28	1.37	3.74	3.62
5A28	2.28	5.40	5.33	5B28	1.57	4.17	4.41	5C28	1.35	3.99	4.17
6A28	2.23	6.13	6.50	6B28	1.45	4.66	4.54	6C28	1.42	4.17	3.99
7A28	2.33	5.64	5.52	7B28	1.30	3.43	3.99	7C28	1.37	3.99	3.80
8A28	2.38	6.32	6.13	8B28	1.18	3.49	3.68	8C28	1.26	3.86	3.92
9A28	2.11	5.33	5.52	9B28	1.42	3.43	3.80	9C28	1.32	3.74	3.56
10A28	1.94	5.40	5.58	10B28	1.30	3.56	3.74	10C28	1.37	4.11	3.86
11A28	2.11	5.52	5.58	11B28	1.30	3.56	3.68	11C28	1.35	3.19	4.05
12A28	2.18	5.33	5.58	12B28	1.29	3.68	3.62	12C28	1.42	4.17	4.23
13A28	1.96	5.15	5.21	13B28	1.69	5.15	5.09	13C28	1.69	5.52	5.58
14A28	2.08	5.21	5.40	14B28	1.74	5.15	5.64	14C28	1.84	6.13	5.89
15A28	1.94	5.09	5.09	15B28	1.64	5.27	5.52	15C28	1.86	5.21	5.52
16A28	1.96	5.15	5.03	16B28	1.74	5.40	5.40	16C28	1.69	5.46	5.52
17A28	1.96	5.27	5.58	17B28	1.59	5.15	4.66	17C28	1.77	5.27	5.46
18A28	1.79	5.27	5.15	18B28	1.55	5.27	5.15	18C28	1.62	5.21	5.64
19A28	1.69	4.97	4.78	19B28	1.47	4.91	5.15	19C28	1.94	5.89	6.25
20A28	1.90	5.33	5.40	20B28	1.59	5.40	5.15	20C28	1.74	5.64	5.76
21A28	1.89	5.15	5.15	21B28	1.52	5.21	5.15	21C28	1.64	5.21	5.27
22A28	2.04	5.27	5.46	22B28	1.79	5.58	5.40	22C28	1.69	5.52	6.07
23A28	1.89	5.21	5.21	23B28	1.59	5.52	5.27	23C28	1.81	5.03	5.76
24A28	1.84	5.03	4.84	24B28	1.67	5.52	5.52	24C28	1.69	5.52	5.70
**AVG.**	**2.05**	**5.42**	**5.44**		**1.53**	**4.66**	**4.69**		**1.55**	**4.51**	**4.63**
**ST. DEV.**	**0.18**	**0.34**	**0.38**		**0.16**	**0.76**	**0.67**		**0.23**	**0.88**	**0.96**
**CV%**	**8.78**	**6.27**	**6.99**		**10.46**	**16.31**	**14.29**		**14.84**	**19.51**	**20.73**

**Table A3:** Tests on specimens 60 days age (Cube 70 × 70 × 70 and Briquette).

Specimen Number	Compressive Strength 70 × 70 × 70 (N/mm^2^)	Tensile Strength Briquette (N/mm^2^)	Specimen Number	Compressive Strength 70 × 70 × 70 (N/mm^2^)	Tensile Strength Briquette (N/mm^2^)	Specimen Number	Compressive Strength 70 × 70 × 70 (N/mm^2^)	Tensile Strength Briquette (N/mm^2^)
A-Type Mortar			B-Type Mortar			C-Type Mortar		
1A60	6.69	1.51	1B60	6.09	1.11	1C60	5.53	0.98
2A60	6.75	1.20	2B60	5.81	1.01	2C60	6.09	0.98
3A60	6.85	1.35	3B60	5.77	0.96	3C60	5.39	0.92
4A60	6.65	1.59	4B60	5.57	1.13	4C60	5.65	1.02
5A60	6.95	1.53	5B60	5.69	1.07	5C60	6.05	1.00
6A60	6.91	1.29	6B60	5.97	1.09	6C60	5.81	1.10
7A60	6.61	1.28	7B60	5.41	1.16	7C60	6.37	0.94
8A60	6.75	1.35	8B60	5.85	0.81	8C60	5.75	1.24
9A60	7.01	1.47	9B60	5.57	1.01	9C60	5.95	1.00
10A60	6.85	1.50	10B60	5.69	1.16	10C60	5.97	0.94
11A60	6.51	1.43	11B60	5.73	1.09	11C60	6.11	1.04
12A60	6.97	1.56	12B60	5.85	1.04	12C60	6.15	0.96
**AVERAGE**	**6.79**	**1.42**		**5.75**	**1.05**		**5.9**	**1.01**
**ST. DEV.**	**0.16**	**0.13**		**0.19**	**0.10**		**0.28**	**0.09**
**CV%**	**2.36**	**9.15**		**3.30**	**9.52**		**4.75**	**8.91**

**Table A4:** Tests on specimens 60 days age (Cube 40 × 40 × 40 and Prism).

Spec. N.	Ind. Tens. Strength 40 × 40 × 160	Compr. Str. 40 × 40 × 40 (N/mm^2^)	Compr. Str. 40 × 40 × 40 (N/mm^2^)	Spec. N.	Ind. Tens. Strength 40 × 40 × 160	Compr. Str. 40 × 40 × 40 (N/mm^2^)	Compr. Str. 40 × 40 × 40 (N/mm^2^)	Spec. N.	Ind. Tens. Strength 40 × 40 × 160	Compr. Str. 40 × 40 × 40 (N/mm^2^)	Compr. Str. 40 × 40 × 40 (N/mm^2^)
A-Type Mortar				B-Type Mortar				C-Type Mortar			
1A60	2.99	8.89	8.89	1B60	2.26	7.17	7.23	1C60	2.26	5.76	6.13
2A60	2.92	8.58	8.89	2B60	2.24	7.23	6.50	2C60	1.84	6.25	6.07
3A60	2.58	8.40	7.97	3B60	2.37	6.56	6.74	3C60	2.13	6.44	6.99
4A60	3.19	9.07	9.20	4B60	2.23	6.99	6.62	4C60	2.16	6.81	6.99
5A60	2.99	8.58	8.71	5B60	2.15	6.99	6.50	5C60	2.21	7.11	7.11
6A60	2.70	8.34	8.22	6B60	2.23	6.93	6.74	6C60	2.08	6.93	6.62
7A60	2.84	8.34	8.22	7B60	2.24	6.38	7.11	7C60	2.26	6.13	6.74
8A60	2.67	8.83	8.34	8B60	2.35	6.93	6.62	8C60	2.31	5.64	6.74
9A60	2.89	8.28	8.71	9B60	2.29	6.74	7.60	9C60	2.26	7.11	7.23
10A60	2.82	8.22	8.40	10B60	2.16	6.50	6.50	10C60	2.08	6.74	6.68
11A60	2.77	8.58	8.46	11B60	2.05	6.99	7.05	11C60	2.16	6.87	7.48
12A60	3.24	8.89	9.07	12B60	2.11	6.25	6.50	12C60	2.04	6.93	6.87
13A60	2.84	8.46	8.52	13B60	2.43	6.99	6.87	13A60	2.35	6.62	6.81
14A60	2.92	8.15	8.46	146B0	2.53	7.11	7.48	14C60	2.18	6.62	6.99
15A60	2.92	8.34	8.40	15B60	1.26	6.87	7.11	15C60	2.18	6.87	6.25
16A60	2.99	9.01	8.52	16B60	1.79	6.50	7.05	16C60	2.11	5.89	6.25
17A60	2.72	8.15	8.28	17B60	2.38	6.74	7.23	17C60	2.18	6.62	5.89
								18C60	2.33	7.05	6.81
**AVG.**	**2.88**	**8.54**	**8.54**		**2.18**	**6.82**	**6.91**		**2.17**	**6.58**	**6.70**
**ST. DEV.**	**0.17**	**0.30**	**0.33**		**0.29**	**0.29**	**0.36**		**0.12**	**0.46**	**0.43**
**CV%**	**5.90**	**3.51**	**3.86**		**13.30**	**4.25**	**5.21**		**5.53**	**6.99**	**6.42**

**Table A5:** Tests on specimens 180 days age (Cube 70 × 70 × 70 and Briquette).

Specimen Number	Compressive Strength 70 × 70 × 70 (N/mm^2^)	Tensile Strength Briquette (N/mm^2^)	Specimen Number	Compressive Strength 70 × 70 × 70 (N/mm^2^)	Tensile Strength Briquette (N/mm^2^)	Specimen Number	Compressive Strength 70 × 70 × 70 (N/mm^2^)	Tensile Strength Briquette (N/mm^2^)
A-Type Mortar			B-Type Mortar			C-Type Mortar		
1A180	9.84	1.79	1B180	8.65	1.24	1C180	10.04	1.22
2A180	9.39	1.92	2B180	8.94	1.42	2C180	10.00	1.18
3A180	9.59	2.06	3B180	8.69	1.52	3C180	9.80	1.80
4A180	9.80	1.72	4B180	8.45	1.72	4C180	9.80	1.48
5A180	10.29	1.72	5B180	8.78	2.10	5C180	9.55	1.76
6A180	9.84	1.96	6B180	9.14	1.54	6C180	10.82	1.20
7A180	9.10	1.96	7B180	8.94	1.64	7C180	10.16	1.64
8A180	9.18	1.70	8B180	9.02	2.06	8C180	11.02	1.36
9A180	10.37	1.78	9B180	9.06	1.72	9C180	9.67	1.58
10A180	8.94	1.71						
11A180	9.92	1.92						
12A180	9.88	1.78						
**AVERAGE**	**9.67**	**1.83**		**8.85**	**1.66**		**10.10**	**1.47**
**ST. DEV.**	**0.45**	**0.12**		**0.22**	**0.28**		**0.51**	**0.24**
**CV%**	**4.64**	**6.67**		**2.54**	**16.87**		**5.01**	**16.43**

**Table A6:** Tests on specimens 180 days age (Cube 40 × 40 × 40 and Prism).

Spec. N.	Ind. Tens. Strength 40 × 40 × 160	Compr. Str. 40 × 40 × 40 (N/mm^2^)	Compr. Str. 40 × 40 × 40 (N/mm^2^)	Spec. N.	Ind. Tens. Strength 40 × 40 × 160	Compr. Str. 40 × 40 × 40 (N/mm^2^)	Compr. Str. 40 × 40 × 40 (N/mm^2^)	Spec. N.	Ind. Tens. Strength 40 × 40 × 160	Compr. Str. 40 × 40 × 40 (N/mm^2^)	Compr. Str. 40 × 40 × 40 (N/mm^2^)
A-Type Mortar				B-Type Mortar				C-Type Mortar			
1A180	2.98	12.88	12.63	1B180	2.48	11.63	11.38	1C180	2.80	16.25	16.25
2A180	3.14	13.50	13.13	2B180	2.68	11.75	12.13	2C180	3.04	16.75	16.25
3A180	2.98	13.38	13.38	3B180	2.66	11.63	11.50	3C180	2.40	15.25	15.38
4A180	2.70	13.13	12.88	4B180	2.44	10.38	10.88	4C180	3.12	15.00	15.13
5A180	3.18	13.88	13.88	5B180	2.72	11.88	11.50	5C180	2.82	14.88	14.75
6A180	3.06	13.50	13.38	6B180	2.56	12.00	12.00	6C180	2.78	14.63	14.88
7A180	2.98	13.88	13.13	7B180	2.46	11.38	11.38	7C180	2.64	12.88	13.00
8A180	2.90	13.63	14.13	8B180	2.40	11.25	11.13	8C180	3.00	14.25	13.50
9A180	2.93	13.13	13.25	9B180	2.54	11.75	11.50	9C180	3.02	14.13	13.75
10A180	3.06	13.13	13.63	10B180	2.52	11.38	11.13	10C180	2.70	13.44	13.75
11A180	2.82	13.75	13.75	11B180	2.56	11.38	11.50	11C180	2.94	13.13	13.25
12A180	2.96	13.63	13.25	12B180	2.70	12.00	11.50	12C180	2.90	13.50	13.75
13A180	2.80	14.00	13.63								
14A180	2.90	13.63	13.75								
15A180	2.84	12.88	13.50								
**AVG.**	**2.95**	**13.46**	**13.42**		**2.55**	**11.53**	**11.46**		**2.85**	**14.51**	**14.47**
**ST. DEV.**	**0.13**	**0.36**	**0.39**		**0.11**	**0.44**	**0.35**		**0.20**	**1.20**	**1.13**
**CV%**	**4.40**	**2.69**	**2.94**		**4.16**	**3.84**	**3.03**		**7.09**	**8.30**	**7.79**
