# Correction: Monaco et al. Sustainable Mortars for Application in the Cultural Heritage Field. *Materials* 2021, *14*, 598

**DOI:** 10.3390/ma14112882

**Published:** 2021-05-27

**Authors:** Michelina Monaco, Marianna Aurilio, Anna Tafuro, Mariateresa Guadagnuolo

**Affiliations:** 1 Department of Engineering, University of Sannio, Piazza Roma 21, 82100 Benevento, Italy; monaco@unisannio.it; 2 Department of Architecture and Industrial Design, University of Campania Luigi Vanvitelli, Abbazia di San Lorenzo ad Septimum, Via S. Lorenzo, 81031 Aversa, Italy; marianna.aurilio@unicampania.it (M.A.); anna.tafuro@unicampania.it (A.T.)

In the initial article, the data in the Appendix A Section, Table A6, were incorrect [1].

## Appendix A

**Table A6:** Tests on 180-day-old specimens (Cube 40 × 40 × 40 and Prism).

Spec. N.	Ind. Tens. Strength 40 × 40 × 160	Compr. Str. 40 × 40 × 40 (N/mm^2^)	Compr. Str. 40 × 40 × 40 (N/mm^2^)	Spec. N.	Ind. Tens. Strength 40 × 40 × 160	Compr. Str. 40 × 40 × 40 (N/mm^2^)	Compr. Str. 40 × 40 × 40 (N/mm^2^)	Spec. N.	Ind. Tens. Strength 40 × 40 × 160	Compr. Str. 40 × 40 × 40 (N/mm^2^)	Compr. Str. 40 × 40 × 40 (N/mm^2^)
A-Type Mortar				B-Type Mortar				C-Type Mortar			
**1A180**	3.72	12.88	12.63	**1B180**	3.10	11.63	11.38	**1C180**	3.50	16.25	16.25
**2A180**	3.92	13.50	13.13	**2B180**	3.35	11.75	12.13	**2C180**	3.80	16.75	16.25
**3A180**	3.72	13.38	13.38	**3B180**	3.32	11.63	11.50	**3C180**	3.00	15.25	15.38
**4A180**	3.37	13.13	12.88	**4B180**	3.05	10.38	10.88	**4C180**	3.90	15.00	15.13
**5A180**	3.97	13.88	13.88	**5B180**	3.40	11.88	11.50	**5C180**	3.52	14.88	14.75
**6A180**	3.82	13.50	13.38	**6B180**	3.20	12.00	12.00	**6C180**	3.47	14.63	14.88
**7A180**	3.72	13.88	13.13	**7B180**	3.07	11.38	11.38	**7C180**	3.30	12.88	13.00
**8A180**	3.62	13.63	14.13	**8B180**	3.00	11.25	11.13	**8C180**	3.75	14.25	13.50
**9A180**	3.66	13.13	13.25	**9B180**	3.17	11.75	11.50	**9C180**	3.77	14.13	13.75
**10A180**	3.82	13.13	13.63	**10B180**	3.15	11.38	11.13	**10C180**	3.37	13.44	13.75
**11A180**	3.52	13.75	13.75	**11B180**	3.20	11.38	11.50	**11C180**	3.67	13.13	13.25
**12A180**	3.70	13.63	13.25	**12B180**	3.37	12.00	11.50	**12C180**	3.62	13.50	13.75
**13A180**	3.50	14.00	13.63	—	—	—	—	—	—	—	—
**14A180**	3.62	13.63	13.75	—	—	—	—	—	—	—	—
**15A180**	3.55	12.88	13.50	—	—	—	—	—	—	—	—
**AVG.**	**3.68**	**13.46**	**13.42**	—	**3.19**	**11.53**	**11.46**	—	**3.56**	**14.51**	**14.47**
**ST. DEV.**	**0.16**	**0.36**	**0.39**	—	**0.13**	**0.44**	**0.35**	—	**0.26**	**1.20**	**1.13**
**CV%**	**4.40**	**2.69**	**2.94**	—	**4.20**	**3.84**	**3.03**	—	**7.09**	**8.30**	**7.79**
